# TCPs, WUSs, and WINDs: families of transcription factors that regulate shoot meristem formation, stem cell maintenance, and somatic cell differentiation

**DOI:** 10.3389/fpls.2014.00427

**Published:** 2014-09-03

**Authors:** Miho Ikeda, Masaru Ohme-Takagi

**Affiliations:** ^1^Division of Strategic Research and Development, Graduate School of Science and Engineering, Satitama UniversitySaitama, Japan; ^2^Research Institute of Bioproduction, National Institute of Advanced Industrial Science and TechnologyTsukuba, Japan

**Keywords:** cell differentiation, transcription factor, activator, repressor

## Abstract

In contrast to somatic mammalian cells, which cannot alter their fate, plant cells can dedifferentiate to form totipotent callus cells and regenerate a whole plant, following treatment with specific phytohormones. However, the regulatory mechanisms and key factors that control differentiation-dedifferentiation and cell totipotency have not been completely clarified in plants. Recently, several plant transcription factors that regulate meristem formation and dedifferentiation have been identified and include members of the TEOSINTE BRANCHED1/CYCLOIDEA/PROLIFERATING CELL FACTOR (TCP), WUSCHEL (WUS), and WOUND INDUCED DEDIFFERENTIATION (WIND1) families. WUS and WIND positively control plant cell totipotency, while TCP negatively controls it. Interestingly, TCP is a transcriptional activator that acts as a negative regulator of shoot meristem formation, and WUS is a transcriptional repressor that positively maintains totipotency of the stem cells of the shoot meristem. We describe here the functions of TCP, WUS, and WIND transcription factors in the regulation of differentiation-dedifferentiation by positive and negative transcriptional regulators.

## INTRODUCTION

Generally, differentiated mammalian cells cannot alter their fate or dedifferentiate to acquire pluripotency. Therefore, the technology to produce iPS (induced Pluripotent Stem) cells by expressing specific transcription factors represents a significant breakthrough for animal research (Takahashi and Yamanaka, 2006). In contrast to mammalian cells, plant cells can alter their cell fate and differentiated somatic cells easily dedifferentiate to form masses of totipotent cells, called callus, following treatment with the phytohormones auxin and cytokinin. A single callus cell can regenerate a whole plant, as shown by carrot somatic embryogenesis (Nomura and Komamine, 1986).

Recent work has identified several key transcription factors that induce cell dedifferentiation. These include members of the TEOSINTE BRANCHED1/CYCLOIDEA/PROLIFERATING CELL FACTOR (TCP), WUSCHEL (WUS), and WOUND INDUCED DEDIFFERENTIATION (WIND) families. TCP transcription factors determine the region where meristem forms during embryogenesis, and thus play a pivotal role in pattern formation (Koyama et al., 2007, 2010). WUSs function in maintenance of stem cell populations in shoot meristems (Laux et al., 1996). WINDs are involved in repair of wound tissues in plants by controlling cell dedifferentiation (Iwase et al., 2011). Analyses of these transcription factors are gradually elucidating the molecular mechanisms that control differentiation and dedifferentiation of plant cells, showing that these mechanisms involve a fine balance of the activities of positive and negative regulators.

In this mini review, we describe the functional roles of TCP, WUS and WIND transcription factors in the control of plant cell differentiation and the molecular mechanisms of differentiation-dedifferentiation, as regulated by positive and negative transcriptional regulators.

## TCP TRANSCRIPTION FACTORS FUNCTION AS NEGATIVE REGULATORS OF MERISTEM FORMATION

The TCP family transcription factors are plant-specific and contain a conserved DNA binding domain, termed the TCP domain. TCP binds the core motif GGnCC (Kosugi and Ohashi, 2002). TCP transcription factors were identified by analysis of mutants that affect various aspects of plant development (Luo et al., 1996; Doebley et al., 1997; Kosugi and Ohashi, 1997; Cubas et al., 1999). For example, the *cincinnata* (*cin*) mutant of *Antirrhinum majus*, which encodes an ortholog of *Arabidopsis thaliana* TCP3 or TCP4, exhibits abnormal curvature of leaves and petals (Nath et al., 2003; Crawford et al., 2004). In *Arabidopsis*, the miR319 (JAW) targets *TCP2, TCP3, TCP4, TCP10* and *TCP24*, and the ectopic expression of miR319/JAW results in a *cin*-like phenotype (Palatnik et al., 2003).

The *Arabidopsis* genome contains 24 genes encoding TCP transcription factors in two subfamilies, CYC/TB and PCF (Cubas, 2000). Analysis of knockout and knockdown mutants has provided limited information on the biological functions of TCP transcription factors, probably due to functional redundancy. However, application of chimeric repressor gene silencing technology (CRES-T) has provided additional clarification of TCP functions. The CRES-T gene silencing system creates a chimeric repressor by fusing a transcriptional activator (or other DNA-binding protein) to the plant-specific SRDX repression domain. This chimeric repressor dominantly suppresses the target genes of the transcription factor, functioning epistatically to any endogenous and functionally redundant transcription factors. As a result, the transgenic plants that express the chimeric repressor exhibit a phenotype similar to loss-of-function mutants of the transcription factor (Hiratsu et al., 2003).

Expression of the TCP3 chimeric repressor (*P35S:TCP3SRDX*) induced abnormal curvature of leaves similar to *P35S:JAW* plants, indicating that the phenotype of *P35S:TCP3SRDX* plants reflects that of loss of function of TCPs (Koyama et al., 2007). The *P35S:TCP3SRDX* lines with strong phenotypes exhibit ectopic formation of meristems on cotyledons, while the ectopic expression of a mutated *TCP3,* which lacks the target site for miR319, suppresses meristem formation, indicating that TCP3 negatively regulates meristem formation (Koyama et al., 2007). One of the targets of TCPs is *CUP-SHAPED COTYLEDON1*, which is the key factor that determines the boundary region where the meristem forms (Aida et al., 1997). *P35S:TCP3SRDX* plants ectopically express *CUC1*, showed that TCP transcription factors suppress the formation of meristem via the negative regulation of the expression of *CUC* genes (Koyama et al., 2007). However, TCP3 acts as a transcriptional activator; therefore, TCP3 might activate the expression of the genes for regulators that suppress the expression of *CUC*s. Several target genes of TCP3 have been identified (Koyama et al., 2010), and include *miR164, ASYMMETRIC LEAVES1* (*AS1*),*INDOLE-3-ACETIC ACID3/SHORT HYPOCOTYL2* (*IAA3/SHY2*) and*SMALL AUXIN UP RNA* (*SAUR*). *AS1* and *IAA* encode regulators of leaf development and auxin signaling, respectively (Byrne et al., 2000; Weijers et al., 2005). *SAUR* is an auxin-inducible gene (Hagen and Guilfoyle, 2002) but its function has not been identified. miR164 targets *CUC1, CUC2,* and neighboring NAC genes (Nikovics et al., 2006; Larue et al., 2009).

**FIGURE 1:**
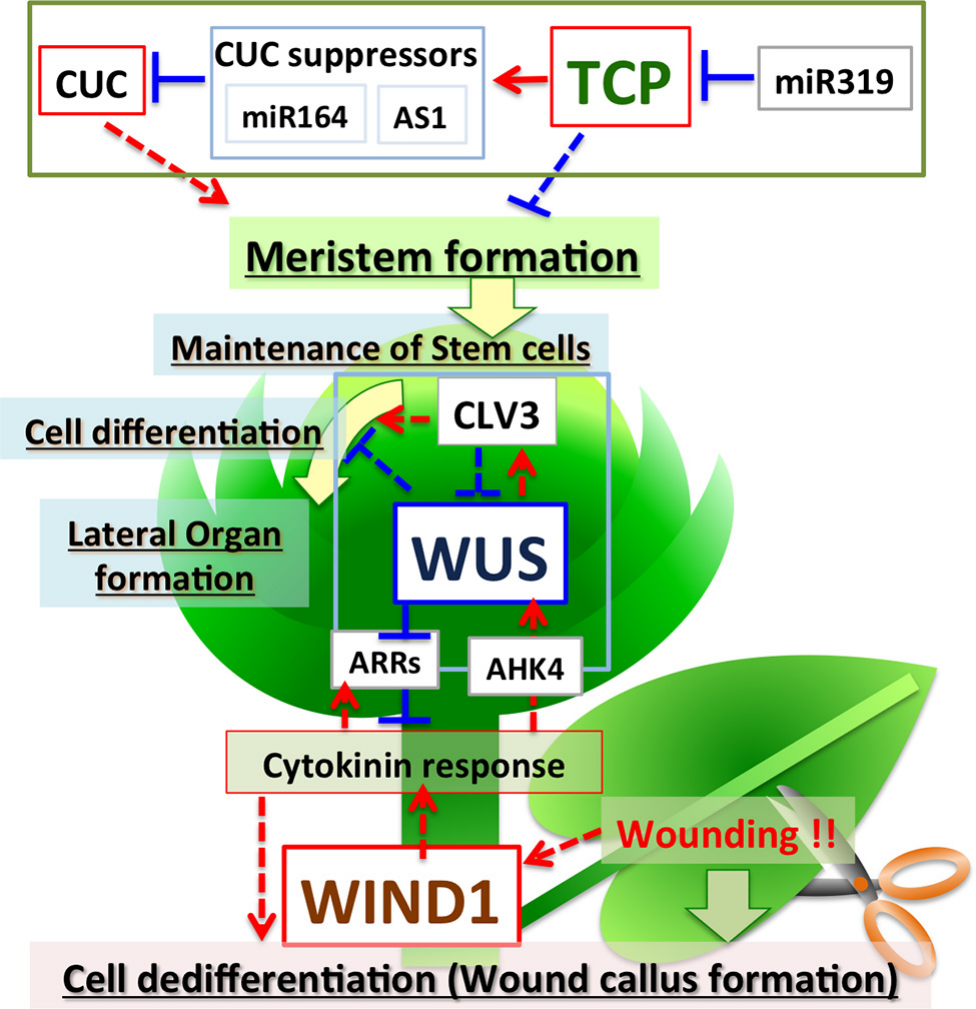
**Regulation of plant cell fate by TCPs, WUS, and WINDs.** TCPs negatively regulate meristem formation *via* direct activation of miRNA164 and AS1, which repress *CUC*s. miR319 negatively regulates expression of *TCP*s. *WUS* encodes a transcriptional repressor, but positively regulates cytokinin signaling by suppressing the expression of *ARRs* to maintain the stem cell population in the shoot meristem. Feedback regulation between WUS and CLVs fine-tunes the maintenance of stem cell populations and meristem size. WINDs are induced by wounding, activate cytokinin signaling, and positively regulate de-differentiation to promote callus formation in wounded tissues. Red arrows indicate positive regulation and blue lines indicate negative regulation. Solid lines indicate direct regulation and dotted lines indicate indirect regulation.

The *TCP* genes are ubiquitously expressed, except in the meristem, and suppress meristem formation. In the region where the shoot apical meristem is formed, miR319 suppresses *TCP* expression, and TCPs activate some suppressor genes including miR164; this results in meristem formation by induction of the expression of *CUC*s (**Figure 1**). Therefore, TCPs play an important role in pattern formation by suppressing the formation of ectopic meristem.

## WUS MAINTAINS STEM CELL POPULATIONS

WUSCHEL, a HOMEOBOX family transcription factor, plays a central role in the maintenance of stem cell populations in shoot meristems (Laux et al., 1996; Mayer et al., 1998; Veit, 2004). In loss-of-function *WUS* (*wus-1*) mutants, new stem cells do not form in the shoot meristem and the meristem of *wus-1* plants stops growing after forming several leaves (Laux et al., 1996). By contrast, ectopic expression of *WUS* increases the size of shoot meristems and induces ectopic cell dedifferentiation, with resultant formation of adventitious shoots and somatic embryos in root tissues (Zuo et al., 2002; Gallois et al., 2004). These results indicate that WUS positively regulates the size of the shoot meristem by maintaining the appropriate number of pluripotent stem cells. WUS acts as a positive regulator of the expression of *CLV3,* which encodes a small peptide ligand that negatively regulates meristem size by suppressing the expression of *WUS* (Schoof et al., 2000; Reddy, 2008). Therefore, feedback regulation between WUS and CLVs finely tunes the size of the meristem (**Figure 1**). WUS functions as a transcriptional repressor (Ikeda et al., 2009), and thus appears to suppress the expression of a negative regulator of *CLV3*.

The *Arabidopsis* WUS family consists of 15 members, *WUS* and the *WUSCHEL-RELATED HOMEOBOX* (*WOX*) genes (Haecker et al., 2004). The WUS family transcription factors contain a conserved WUS-box domain in addition to the homeodomain (Haecker et al., 2004). The WUS-boxes of WUS and 7 WOXs also contain a TLXLFP motif, which functions in repression of transcription (Ikeda et al., 2009). WUS proteins with a mutation in the TLXLFP motif lost activity for maintenance of stem cell population and induction of cell dedifferentiation, and SRDX repression domain is able to complement loss of TLXLFP motif activity in the mutated WUS protein, indicating that the maintenance of stem cell population and induction of cell dedifferentiation require the repressive activity of WUS.

*ARABIDOPSIS RESPONSE REGULATOR7* (*ARR7*), a type A response regulator, negatively regulates cytokinin signaling and suppresses the size of the meristem (Leibfried et al., 2005; Zhao et al., 2010). WUS directly binds to the 5′ region upstream of the *ARR7* gene to suppress its expression (**Figure 1**). Because ARR7 negatively regulates the size of the meristem, suppression of *ARR7* by WUS might be important to maintain stem cell populations. The expression of *ARR7* and *ARR15* is regulated by auxin *via* activation of AUXIN RESPONSE FACTOR5/MONOPTEROS (Leibfried et al., 2005; Zhao et al., 2010). The expression of *ARRs* and *WUS* is positively regulated by cytokinin (**Figure 1**) (Holt et al., 2014). These observations suggest that type A response regulators regulate differentiation–dedifferentiation of plant cells mediated by WUS, auxin and cytokinin. In addition to *ARR*s, *TOPLESS, CLV1, KANADI1, KANADI2, ASYMMETRIC LEAVES2,* and*YABBY*, which are involved in cell differentiation and leaf development, are also direct targets of WUS (Busch et al., 2010; Yadav et al., 2013). WUS may maintain meristematic pluripotent stem cells by suppressing the expression of these genes related to cell differentiation.

## WINDs REGULATE CELL DEDIFFERENTIATION DURING THE WOUNDING RESPONSE

Similar to other multicellular organisms, plants regenerate new organs to repair wounded tissues. In wound repair, somatic cells of wound tissues first dedifferentiate to form a mass of pluripotent cells called callus. Then the callus cells re-differentiate and regenerate the organ. Wound-induced cell dedifferentiation is commonly observed in various multicellular organisms and several key factors that induce meristem formation have been identified (Stappenbeck and Miyoshi, 2009), but the molecular mechanisms that induce the wounded cells into dedifferentiated status during wound repair have not been clarified in plants.

Recent work showed that AP2/ERF transcription factors WIND1, WIND2, WIND3, and WIND4 function as master regulators that control dedifferentiation in plants (Iwase et al., 2011). Comparison of gene expression between *Arabidopsis callus* and seedlings revealed that *WIND1* is specifically expressed in callus. *WIND1* is also rapidly induced after wounding and specifically expressed in the wound site. Remarkably, *Arabidopsis* plants that ectopically express *WIND1* (*P35S:WIND1*) form callus after germination. The *P35S:WIND1* callus has similar expression profile to the callus induced by auxin and cytokinin. The expression of *WIND1* alone is sufficient to induce cell dedifferentiation to form callus and to maintain callus without auxin or cytokinin; therefore, WIND1 functions as a master regulator of cell dedifferentiation in *Arabidopsis*. Interestingly, *P35S:WIND1* callus does not show increased auxin content or increased activity of the auxin reporter *DR5*. By contrast, the *P35S:WIND1* callus does show increased cytokinin content. *P35S:WIND1* enhances callus formation at low cytokinin concentrations, concentrations that do not induce callus production in wild-type *Arabidopsis* plants. Also, *arr1 arr2* mutants, which are defective for type-B ARR-mediated cytokinin signaling, suppress callus formation by *P35S:WIND1.* These observations suggest that WIND1 induces callus formation by activating cytokinin signaling, but not auxin signaling (**Figure 1**).

## CONCLUSIONS AND FUTURE PROSPECTS

Recent work has identified factors that regulate differentiation-dedifferentiation of plant cells. TCP, WUS, and WIND transcription factors are involved in the regulation of differentiation of plant cells, but each of these transcription factor families has different molecular functions and different roles in controlling cell fate. The *TCP* genes are highly conserved among plant genomes and form a multigene family with pivotal roles in plant development. TCPs are transcriptional activators, but act as negative regulators of cell dedifferentiation and suppress meristem formation *via* activation of miRNA164 and AS1 to repress the expression of the *CUC* genes. *TCP*s are also negatively regulated by miR319. By contrast, WUS is a transcriptional repressor, but acts as a positive regulator promoting cell dedifferentiation. One of the direct targets of WUS is a type A response regulator, *ARR7,* which acts as a suppressor of cytokinin signaling. WUS positively regulates cytokinin signaling by suppressing the expression of *ARR7*. Maintenance of stem cell populations and meristem size are fine-tuned by feedback regulation between WUS and CLVs. WINDs are transcriptional activators and promote dedifferentiation similar to WUS. WINDs appear to activate cytokinin signaling. Thus, positive regulators and various types of negative regulators control cell differentiation and dedifferentiation. In addition, it is typical in plants that transcriptional repressors (WUS) positively regulate dedifferentiation, and transcriptional activators (TCP) negatively regulate dedifferentiation. Fine tuning systems *via* suppression of a negative regulator by another negative regulator, thus resulting in positive regulation, appears to be employed in the regulation of cell differentiation-dedifferentiation in plants.

As a future step, it will be necessary to identify all the factors that positively and negatively regulate cell differentiation and the signaling networks that are regulated by those factors. Because the molecular mechanisms of dedifferentiation by auxin and cytokinin have not been fully identified, further work will also involve detailed analysis of WIND, WUS, and TCP functions. Control of totipotency of plant cells is also important for breeding, production of new cultivars, and genetic engineering.

## Conflict of Interest Statement

The authors declare that the research was conducted in the absence of any commercial or financial relationships that could be construed as a potential conflict of interest.
